# Semantics-Powered Healthcare Engineering and Data Analytics

**DOI:** 10.1155/2017/7983473

**Published:** 2017-10-26

**Authors:** Zhe He, Cui Tao, Jiang Bian, Michel Dumontier, William R. Hogan

**Affiliations:** ^1^School of Information, Florida State University, Tallahassee, FL, USA; ^2^School of Biomedical Informatics, University of Texas Health Science Center at Houston, Houston, TX, USA; ^3^Department of Health Outcomes and Policy, University of Florida, Gainesville, FL, USA; ^4^Maastricht University, Maastricht, Netherlands

## 1. Introduction

Health information systems (HISs) play a crucial role in healthcare in the 21st century. Enacted as part of the American Recovery and Reinvestment Act of 2009, the Health Information Technology for Economic and Clinical Health (HITECH) Act was signed into law on February 17, 2009, to promote the adoption and meaningful use of health information technology. Since then, the percentage of non-United-States-federal acute care hospitals that adopted basic electronic health record (EHR) systems increased from 9.4% in 2008 to 75.5% in 2014 [1]. However, even though the adoption of EHRs by hospitals is successful in the United States, the interoperability of the HISs is falling behind, hampering data exchange among healthcare organizations and meaningful aggregation of heterogeneous healthcare data. As such, even though the dramatically increasing amount of healthcare data offers unprecedented opportunities for mining previously unknown knowledge, it is still challenging to properly leverage data from different sources, to overcome barriers in data analytics (e.g., missing data), and to solve real world problems such as early diagnosis of medical conditions, prediction of disease progression, and identification of patient cohorts for clinical trials.

Biomedical ontologies and controlled vocabularies provide structured domain knowledge to support data standardization and interoperability in a variety of HISs such as EHR, healthcare administration, and clinical decision support. Some notable examples include the International Classification of Diseases (ICD), which is a widely used controlled vocabulary for encoding billing diagnoses and procedures in HISs [2]. SNOMED CT is used for encoding problem lists in EHRs [3]. RxNorm is a medical terminology that normalizes names of all clinical drugs available on the United States market and links to many of the drug vocabularies commonly used in pharmacy management [4]. Moreover, with rich concepts linked by semantic relationships, they have also been widely used in natural language processing, data mining, machine learning, semantic annotation, and automated reasoning. The Unified Medical Language System (UMLS), developed and maintained by the United States National Library of Medicine, is a compendium of over 190 controlled vocabularies and biomedical ontologies, including ICD, SNOMED CT, and RxNorm. In the renaissance of artificial intelligence (AI), knowledge-intensive and/or data-driven medical applications and research will directly benefit from the formalisms and rich knowledge encoded in the biomedical ontologies. However, they have not yet been fully capitalized in the healthcare engineering and data analytics.

The main goal of this special issue is to bring together the researchers in the field of knowledge representation, data management, and health data analytics to introduce innovative semantics-based methods to address important problems in healthcare engineering, illustrate the use of biomedical ontologies and semantic web technologies to discover hidden knowledge in biomedical and health data, and showcase state-of-the-art research and development. The selected papers underwent a rigorous review and revision process. We are glad to see that the selected papers presented novel usage of semantics-based techniques and ontologies in pressing health-related problems such as healthcare data integration, pattern mining from EHRs, medical entity recognition in clinical text, and clinical data sharing. Meanwhile, we have also seen foundational research papers that are focused on developing and curating biomedical ontologies. The multidisciplinary nature of this special issue is reflected by the fact that the problems in semantics-based healthcare engineering and data analytics are being tackled by researchers in different communities, including computer science, medicine, biomedical engineering, biomedical informatics, statistics, and so forth.

## 2. Papers in This Special Issue

In this special issue, we present 8 novel studies about semantics-powered healthcare engineering and data analytics. These studies can be categorized into the following three topics: (1) natural language processing and data mining, (2) clinical data sharing and data integration, and (3) ontology engineering and quality assurance (QA).

### 2.1. Natural Language Processing and Data Mining

Natural language processing (NLP), which can unlock knowledge and detailed information from semistructured or unstructured medical data (e.g., clinical narratives in EHRs and pathology reports), has been widely used to support outcome reporting, clinical research, and operations. However, the free format of clinical text, which may contain acronyms (e.g., COPD, ADR, and BP), typographical errors, and polysemy (e.g., cold), poses significant challenges in text processing and understanding. Basic NLP tasks such as named entity recognition (NER) and word sense disambiguation (WSD) have been widely studied in alphabetic languages such as English. The abundance of controlled vocabularies in English also eases the task of NER of English text. In character-based languages such as Chinese with no space between words and few controlled vocabularies, word segmentation is a particularly difficult problem. In the article titled “A Novel Approach towards Medical Entity Recognition in Chinese Clinical Text,” using the Chinese drug name dictionaries, J. Liang et al. propose a cascade-type Chinese medication entity recognition approach that aims at integrating the sentence category classifier using a support vector machine and the conditional random field-based medication entity recognition. They applied this technique on a test set of 324 Chinese-written admission notes with manual annotation by medical experts and showed promising results.

Automated text classification has been a popular application of NLP. When dealing with a large amount of text such as online forum postings, traditional manual text classification has a significant limitation with respect to scalability. In the article, “An Interpretable Classification Framework for Information Extraction from Online Healthcare Forums,” J. Gao et al. introduced an innovative and effective random forest-based model with interpretable results for classifying sentences in online healthcare forum posts into three classes: medication, symptom, and background. The features used to train the model include labeled sequential patterns, UMLS semantic types, sentence-based features, and heuristic features. This approach can potentially help researchers and clinicians better understand and analyze patients' opinions and needs toward various health topics.

To make an ontology useful for automated term extraction from text, it is important to assess its coverage. In the article entitled “Semantic Modeling for Exposomics with Exploratory Evaluation in Clinical Context,” J. Fan et al. introduced their research on creating an exposome-oriented semantic network from existing ontology entities and relations. They then evaluated the derived semantic network in terms of literature coverage and text annotation.

Controlled vocabularies and biomedical ontologies can facilitate the task of association patterns mining in the unstructured medical data. In the article entitled “Association Patterns of Ontological Features Signify Electronic Health Records in Liver Cancer,” L. W. C. Chan et al. identified the association patterns for liver cancer patients by extracting terms from liver cancer reports and mapping them to SNOMED CT concepts. They further quantified the association levels between every two features in cases of hepatocellular carcinoma or liver metastases and those with no abnormality detected.

### 2.2. Clinical Data Sharing and Data Integration

With the increasing adoption of EHRs and various HISs in the healthcare organizations, health data are generated in an unprecedented speed and amount. Data sharing and data integration can mitigate the biases of disparate data sources to support more meaningful data analytics. Towards this end, national consortiums in the United States such as the eMERGE (Electronic Medical Records and Genomics) Network, Clinical and Translational Science Institutes, Patient-Centered Clinical Research Network (PCORnet), and Observational Health Data Sciences and Informatics (OHDSI) are putting concerted efforts into creating data models, resources, and tools to support sharing and integration of healthcare data from heterogeneous sources. Such efforts are also being made in other countries such Italy and Thailand. In the article “A SOA-Based Platform to Support Clinical Data Sharing,” R. Gazzarata et al. introduced a service-oriented architecture-based platform to support technical interoperability. The platform uses Health Level Seven (HL7) Version 3 messages combined with the LOINC (Logical Observation Identifiers Names and Codes) vocabulary to ensure semantic interoperability among HISs in Italy. In the article titled “Graph-Based Semantic Web Service Composition for Healthcare Data Integration,” N. Arch-int et al. proposed a composition system based on semantic web services to integrate healthcare data in different health organizations in Thailand and evaluated the system with respect to execution time and correctness.

### 2.3. Ontology Engineering and Curation

Without a well-curated metadata standard, large health datasets are hard to manage and analyze. Efforts have been made to develop metadata standards, often in the form of ontologies, to organize large health data set in a semantic knowledge base. In their article “A Granular Ontology Model for Maternal and Child Health Information System,” S. Ismail et al. presented a data access model for managing maternal and child health data leveraging Fast Healthcare Interoperability Resources (FHIR), the latest data exchange standard created by HL7. They targeted completeness of maternal and child-related health information systems in developing countries.

Due to the size and complexity of biomedical ontologies, modeling errors, missing concepts, missing relationships, and inconsistencies are inevitable, limiting their utility in critical clinical applications and biomedical research. Automated and semiautomated QA methods, which can highlight the errors in an ontology, will lead to high QA yields and better utilization of QA personnel. In their article “Taxonomy-Based Approaches to Quality Assurance of Ontologies,” M. Halper et al. presented a guideline for choosing and combining appropriate abstraction networks for an ontology to automatically identify sets of concepts that are expected to have a high likelihood of errors.

## 3. Discussion and Conclusion

In the big data era, the vast amount of healthcare data poses significant challenges in data management and analysis. Semantics-based knowledge representations and methods, which encapsulate structured domain knowledge, play an important role in overcoming these challenges. As shown in this special issue, important problems in data analytics including data mining, natural language processing, data sharing, data integration, and ontology engineering are being tackled by multidisciplinary teams with diverse expertise. Moreover, new methods, platforms, and algorithms have been developed to integrate, process, and analyze diverse types of health data and transform them into actionable knowledge and wisdom for better patient care and clinical practice. We envision this work will have significant impact in healthcare engineering and data analytics. We are looking forward to seeing more work in this area that is motivated by this special issue.
